# Glyphosate-Resistant *Parthenium hysterophorus* in the Caribbean Islands: Non Target Site Resistance and Target Site Resistance in Relation to Resistance Levels

**DOI:** 10.3389/fpls.2016.01845

**Published:** 2016-12-06

**Authors:** Enzo Bracamonte, Pablo T. Fernández-Moreno, Francisco Barro, Rafael De Prado

**Affiliations:** ^1^Faculty of Agricultural Sciences, National University of Córdoba (UNC)Córdoba, Argentina; ^2^Department of Agricultural Chemistry and Edaphology, University of CordobaCordoba, Spain; ^3^Department of Plant Breeding, Institute for Sustainable Agriculture (IAS), Spanish National Research Council (CSIC)Cordoba, Spain

**Keywords:** *P. hysterophorus*, target-site and non-target-site mechanisms, resistance levels, glyphosate

## Abstract

Glyphosate has been the most intensely herbicide used worldwide for decades, and continues to be a single tool for controlling weeds in woody crops. However, the adoption of this herbicide in a wide range of culture systems has led to the emergence of resistant weeds. Glyphosate has been widely used primarily on citrus in the Caribbean area, but a study of resistance in the Caribbean islands of Cuba and the Dominican Republic has never been carried out. Unfortunately, *Parthenium hysterophorus* has developed glyphosate-resistance in both islands, independently. The resistance level and mechanisms of different *P. hysterophorus* accessions (three collected in Cuba (Cu-R) and four collected in the Dominican Republic (Do-R) have been studied under greenhouse and laboratory conditions. In *in vivo* assays (glyphosate dose causing 50% reduction in above-ground vegetative biomass and survival), the resistance factor levels showed susceptible accessions (Cu-S ≥ Do-S), low-resistance accessions (Cu-R3 < Do-R4), medium-resistance accessions (Do-R3 < Cu-R2 < Do-R2) and high-resistance accessions (Do-R1 < Cu-R1). In addition, the resistance factor levels were similar to those found in the shikimic acid accumulation at 1000 μM of glyphosate (Cu-R1 ≥ Do-R1 > Do-R2 > Cu-R2 > Do-R3 > Do-R4 > Cu-R3 >> Cu-S ≥ Do-S). Glyphosate was degraded to aminomethylphosphonic acid, glyoxylate and sarcosine by >88% in resistant accessions except in Cu-R3 and Do-R4 resistant accessions (51.12 and 44.21, respectively), whereas a little glyphosate (<9.32%) was degraded in both susceptible accessions at 96 h after treatment. There were significant differences between *P. hysterophorus* accessions in the 5-enolpyruvylshikimate-3-phosphate synthase (EPSPS) activity enzyme with and without different glyphosate rates. The R accessions showed values of between 0.026 and 0.21 μmol μg^−1^ TSP protein min^−1^ basal EPSPS activity values with respect to the S (0.024 and 0.025) accessions. The same trend was found in the EPSPS enzyme activity treated with glyphosate, where a higher enzyme activity inhibition (glyphosate μM) corresponded to greater resistance levels in *P. hysterophorus* accessions. One amino acid substitution was found at position 106 in EPSPS, consisting of a proline to serine change in Cu-R1, Do-R1 Do-R2. The above-mentioned results indicate that high resistance values are determined by the number of defense mechanisms (target-site and non-target-site resistance) possessed by the different *P. hysterophorus* accessions, concurrently.

## Introduction

Herbicide resistance is an evolutionary phenomenon that allows resistant weed biotypes to be exposed to the normal dose of a herbicide undergoing any suffering growth alterations (Fernández et al., 2016). This biological phenomenon is favored by intensive herbicide applications with the same active ingredient or with the same mode of action (Neve et al., 2014; Evans et al., 2016). Glyphosate weed resistance is one of the world's most interesting cases, 35 glyphosate-resistant species have been detected and characterized (mainly using test dose response curves and shikimic acid accumulation) up to date (Heap, 2016).

Glyphosate ((N-phosphonomethyl)-glycine) is a post-emergent herbicide that is non-selective, highly systemic and widely used for weed control around the world (Franz et al., 1997; Székács and Darvas, 2012). It is well metabolized in plants and slow-acting with visible phytotoxic symptoms in sensitive plants at 10–20 days after application (Amrhein et al., 1980; Shingh and Shaner, 1998; Monquero et al., 2004). It inhibits the shikimate pathway by inhibiting 5-enolpyruvylshikimate-3-phosphate synthase (EPSPS), which catalyzes the synthesis reactions of aromatic amino acids involved in the formation of essential proteins in plants (Sammons and Gaines, 2014).

Glyphosate resistance selection is due to two different mechanisms known as non-target site resistance (NTSR) and target site resistance (TSR) (Shaner et al., 2012; Sammons and Gaines, 2014). NTSR involves a reduced rate of herbicide in the meristem tissues due to limited absorption/translocation, and/or sequestration of the herbicide into compartments such as vacuoles (Michitte et al., 2007; Ge et al., 2012; Vila-Aiub et al., 2012). Metabolic pathways capable of degrading the herbicide to non-toxic compounds in plants also belong to these group mechanisms (De Prado and Franco, 2004; Cruz-Hipólito et al., 2009, 2011; Busi et al., 2011; de Carvalho et al., 2012; González-Torralva et al., 2012; Alcántara-de la Cruz et al., 2016a). TSR has been produced by one or more mutations in the DNA sequence (González-Torralva et al., 2014; Sammons and Gaines, 2014; Fernández et al., 2015; Yu et al., 2015), or by the overexpression of the EPSPS protein by gene amplification (Gaines et al., 2010; Salas et al., 2012, 2015).

When growers reported noticing any deficiency in their weed control, they usually increased the glyphosate doses, which increased the pressure selection as well as triggering the acquisition of a second resistance mechanism (Jasieniuk et al., 1996; González-Torralva et al., 2012). Then, the level of weed resistance to glyphosate increased (Bostamam et al., 2012).

Ragweed parthenium (*Parthenium hysterophorus* L.) is a troublesome annual weed of the *Asteraceae* family that is native to the Gulf of Mexico and other Latin American countries (Rosario et al., 2013). Its prolific seed production (130,000–200,000 seeds m^−2^), as well as the seeds's ability to persist in the soil and germinate over a wide range of temperatures, have contributed to the widespread distribution of ragweed parthenium in perennial and annual crops (orchards, citrus, soybean, corn) as well as in surrounding areas (Joshi, 1991; Pandey et al., 2003; Navie et al., 2004; Adkins and Shabbir, 2013). In addition, the subtropical environment of the Caribbean Islands (Cuba and Dominican Republic) allows year-round germination, growth, and reproduction of ragweed parthenium, which also contributes to its widespread distribution in the region. Glyphosate has been used repeatedly in perennial crop areas and fallow fields in the Caribbean Islands for many years to manage ragweed parthenium and other troublesome weeds. However, growers have recently observed reduced ragweed parthenium control with single or multiple glyphosate applications. Previous reports have documented glyphosate-resistant ragweed parthenium in Colombia (Rosario et al., 2013), Florida (southeast US) (Fernandez, 2013) and Dominican Republic (Jimenez et al., 2014), but in these three cases the causes of resistance to glyphosate have been inconclusive.

The main objective of this work is a survey of *P. hysterophorus* in Cuba and the Dominican Republic that had never been done before. The specific objectives were to determine (1) the level of glyphosate resistance of different accessions; (2) the possible NTSR and TSR mechanisms involved; and (3) to find out if the resistance genes may also increase the multiplicative or additive resistance levels in *P. hysterophorus*.

## Materials and methods

### Plant material

In 2013, mature *P. hysterophorus* seeds were collected from plants not controlled with glyphosate at doses normally used (2 L ha^−1^; 720 g ae ha^−1^) in areas with perennial crops in two Caribbean Islands. Seeds from Cu-S and Do-S accessions never exposed to glyphosate were collected from adjacent areas and used as a reference control (Table 1). Seeds collected from 25 mature plants were stored under laboratory conditions (25°C) for 2 weeks and then placed in paper bags at 4°C. Approximately 300 seeds of these accessions were sown directly into trays (40 × 60 × 15 cm), containing a mixture of sand and peat (2:1, v/v) and placed in a greenhouse at 28/20°C day/night under a 16 h photoperiod with 850 μmol m^−2^ s^−1^ photon flux density, and 80% relative humidity. At the four leaf stage plants of all accessions were treated with glyphosate at 720 g ae ha^−1^ using a laboratory spray chamber equipped with a flat fan nozzle (TeeJet 8002 EVS) with a total output volume of 200 L ha^−1^ water at a pressure of 200 kPa. Four weeks after glyphosate treatment plant survival of the resistant accessions was estimated, and seed produced from surviving plants was collected and stored in paper bags for all subsequent trials. In the case of susceptible accessions (Cu-S and Do-S), no plant survival was observed 4 weeks after glyphosate treatment.

**Table 1:** **History of different *P. hysterophorus* accessions used in this study**.

**Accessions^a^**	**Location**	**Crop**	**Glyphosate^b^ (time of applications per year), number of application years**
Cu-R1	Ceiba	Orchards^c^	720 (2 or 3 times), > 10
Cu-R2	Ceiba	Citrus^c^	720 (1 time), > 10
Cu-R3	Arimao	Citrus	720 (2 times), unknown
Cu-S	Arimao	Road trails	No herbicide treatment
Do-R1	Villa Altagracia	Citrus^c^	900 (2 times), > 15
Do-R2	San Cristobal	Citrus	900 (2 times), > 15
Do-R3	Monseñor Nouel	Citrus	720 (2 times), > 10
Do-R4	Maria T. Sánchez	Orchards	720 (1 time), > 10
Do-S	Maria T. Sanchez	Road trails	No herbicide treatment

a Cu, P. hysterophorus harvested in Cuba; Do, P. hysterophorus harvested in Dominican Republic;

b glyphosate g ae ha^−1^;

c the last application was performed manually for every plant.

### Dose-response assay

Seeds of putative resistant (Cu-R1, Cu-R2, Cu-R3, Do-R1, Do-R2, Do-R3, and Do-R4) and susceptible (Cu-S and Do-S) of the *P. hysterophorus* accessions were germinated in trays (12 × 12 × 6 cm) containing the same substrate as described before and placed in a growth chamber of similar environmental conditions controlled as before. One week after germination, individual seedlings were transplanted into pots (6 × 6 × 8 cm) and grown under fluctuating 30/20°C day/night with a 14 h photoperiod and 850 μmol m^−2^ s^−1^ photon flux density, and 80% relative humidity. As glyphosate (EPSPS inhibitor) is used in early post-emergence, at the four leaf stage, resistant and susceptible *P. hysterophorus* seedlings were treated with increasing glyphosate doses: 0, 31.25, 62.5, 125, 250, 500, 1000, 2000, 4000, and 8000 g ae ha^−1^ (Roundup Energy 45% w/v, SL, Monsanto Spain). The experiment were conducted with 10 replications (one plant pot^−1^) of each accession per herbicide dose, and the experiments were repeated twice. Thirty days after herbicide treatment, herbicide effects on plant survival (LD) and above-ground vegetative biomass (GR) were assessed.

### Leaf segment shikimate accumulation assay

Leaf segments (50 mm diameter) were harvested from the youngest fully expanded leaf from a batch of 15 plants per *P. hysterophorus* accessions at the 4–6 leaf stage (Hanson et al., 2009). Approximately 50 mg of fresh tissue was transferred to 2 mL Eppendorf tubes containing 1 mL of 1 mM NH_4_H_2_PO_4_ (pH 4.4). Glyphosate was added to the tubes at the following concentrations: 0, 0.1, 0.5, 1, 5, 10, 50, 100, 200, 400, 500, 600, and 1000 μM. The Eppendorf tubes were incubated in a growth chamber during 24 h under the previously described conditions. After 24 h, the tubes were stored at −20°C until analysis. Eppendorf tubes were removed from the freezer and thawed at 60°C for 30 min. Two hundred and fifty micro liters of 1.25 N HCL was added to each tube, and placed at 60°C for 15 min. A 125 μL aliquot from each tube was pipetted into a new 2 mL Eppendorf tube, and 500 μL of periodic acid and sodium metaperiodate (0.25% [wt/v] each) was added. They were incubated at room temperature for 90 min, after which 500 μL of 0.6 N sodium hydroxide and 0.22 M sodium sulfite was added. The contents of all tubes were transferred to glass vials. Samples were measured in a spectrophotometer at 380 nm within 30 min. For each glyphosate concentration and accession, three replications were stablished and repeated twice.

### ^14^C glyphosate absorption and translocation

Absorption and translocation study was carried out following the methodology proposed by Cruz-Hipólito et al. (2011) The ^14^C-glyphosate was mixed with commercially formulated glyphosate to prepare a solution with a specific activity of 0.834 kBq μL^−1^ and a glyphosate concentration of 1.8 g ae L^−1^ (360 g ae ha^−1^ in 200 L). *P. hysterophorus* plants at 4-leaf stage were treated with the radiolabeled herbicide by applying one droplet of 1 μL of glyphosate solution (0.834 kBq μL^−1^) on the adaxial surface of the second leaf in each plant using a micropipette (LabMate). The ^14^C-glyphosate unabsorbed in the treated leaf was removed with 3 mL of water: acetone solution (1:1, v/v) 96 h after droplet application. Preliminary assays with two accessions (Cu-R1 and Cu-S) studied had revealed that the glyphosate absorption leveled-off at 96 h after the droplet applications. The rinsate was mixed with 2 mL of scintillation liquid and analyzed by liquid scintillation spectrometry (LSS) (Scintillation Counter, Beckman LS 6500, Fullerton CA). The plants were separated into the treated leaf, rest of the shoot and root after being placed in cellulose cones. The plant tissue was dried at 60°C over 96 h and combusted in a biological sample oxidizer (Packard Tri Carb 307, Perkin-Elmer, Waltham, MA). The ^14^CO_2_ evolved was trapped and counted in 18 mL of a mixture of Carbo-Sarb E and Permafluor (9:9, v/v) (Perkin-Elmer). Thus, over 95% of the total radioactivity applied was recovered. There were five replications and the experiment was arranged in a completely randomized design, and repeated twice. The proportion of absorbed herbicide was expressed as:

$$\begin{array}{l} {}[\%\text{absorbed}=(\text{kBq in combusted tissue}/(\text{kBq in combusted}\\ \text{ tissue }+\text{ kBq in leaf washes}))\times 100]. \end{array}$$

### Glyphosate metabolism

*P. hysterophorus* plants were treated with a glyphosate rate of 360 g ae ha^−1^ at 4–6 leaf stage. At 96 h after treatment (HAT), glyphosate and its metabolites, i.e., AMPA (aminomethylphosphonic acid), glyoxylate and sarcosine, were determined by reversed-polarity capillary electrophoresis following the methodology described by Rojano-Delgado et al. (2010). The calibration equations were established using non-treated plants and known concentrations of glyphosate and its metabolites, which were determined from their enclosed areas under the peaks in the electropherogram. The average value for the amount of glyoxylate naturally produced by the plant was subtracted from the average of the produced or reduced amount after treatment of each accession (Rojano-Delgado et al., 2010). The experiment was arranged in a completely randomized design with four replications per accession and repeated three times.

### EPSPS enzyme activity assays

The enzyme extraction was conducted according to the protocol described by Dayan et al. (2015). Five gram of the leaf tissue of all *P. hysterophorus* accessions (Table 1) were ground to fine powder in a chilled mortar. Immediately after that, the powdered tissue was transferred to tubes containing 100 mL of cold extraction buffer (100 mM MOPS, 5 mM EDTA, 10% glycerol, 50 mMKCl and 0.5 mM benzamidine) containing 70 μL of β-mercaptoethanol and 1% in polyvinylpolypyrrolidone (PVPP). Samples were stirred and subsequently centrifuged for 40 min (18,000 g) at 4°C. The supernatant was decanted into a beaker using a cheesecloth. (NH_4_)_2_SO_4_ was added to the solution to obtain 45% (w/v) concentration, with stirring during 30 min. After that, the mix was centrifuged at 20,000 g for 30 min at 4°C. The previous step was repeated to precipitate the protein in the extracts but in that case with a (NH_4_)_2_SO_4_ concentration of 80% (w/v) stirring for 30 min. Finally, they were centrifuged at 20,000 × g for 30 min at 4°C.

All the pellets were dissolved in 3 mL of extraction buffer and dialyzed in 2 L of dialysis buffer (30 mm, 1000-MWC dialysis tubing at 4°C on a stir plate) over 12 h. The protein concentrations were determined by Bradford assay (Bradford, 1976).

The assay for the determination of EPSPS activity followed the methodology described by Dayan et al. (2015) using the EnzCheck phosphate assay Kit (Invitrogen, Carlsbad, CA) to determine the inorganic phosphate release. The EPSPS activity from the nine accessions was determined in the presence and absence of glyphosate. The glyphosate concentrations used were: 0, 0.1, 1, 10, 100, and 1000 μM to determine the enzyme activity inhibition (I_50_). The assay buffer was composed of 1 mM MgCl_2_, 10% glycerol, and 100 mM MOPS, 2 mM sodiummolybdate and 200 mM NaF. The experiments were conducted with three replications of each accession per glyphosate concentration and repeated three times. EPSPS enzyme activity was expressed as percentage of enzyme activity in presence of glyphosate respect to the control (without glyphosate).

### EPSP synthase gene sequencing

For RNA extraction 100–200 mg of young leaves were taken from plants of each *P. hysterophorus* accession, and stored at −80°C for the extraction of RNA. Their tissue was ground in liquid nitrogen in a STAR-BEATER 412–0167 mill (VWR International Eurolab S.L., Barcelona, Spain). Total RNA was isolated from leaves as described by Pistón (2013), and the amount and quality were determined in a NanoDrop ND-1000 spectrophotometer (Thermo Scientific, Walthman, MA, USA). The synthesis to cDNA was from total RNA being adjusted to the same concentration in all the samples (50 ng μL^−1^). An iScript^TM^ cDNA Synthesis Kit (Bio-Rad Laboratories, Inc. CA, USA) at 40 μL reaction volume was used following the manufacturer‘s instructions.

The PCR reactions were carried out with cDNA samples from each of the accession using the primers *Bidens*-F10 (5′- GGTTGTGGYGGTVTRTTTCC-3′) and *Bidens*-R11 (5′- GTCCCAASTATCACTRTGTTC-3′) based on EPSPS gene sequences described previously (Alcántara-de la Cruz et al., 2016b). PCR conditions were also as described (Alcántara-de la Cruz et al., 2016b). The PCR on cDNA amplified fragments of 462 bp in length, comprising the region of Thr-102 and Pro-106, which corresponds to the sequence of the EPSPS gene of *Arabidopsis* Klee et al. (1987), in which point mutations conferring resistance to glyphosate have been associated (Sammons and Gaines, 2014; Yu et al., 2015).

The PCR fragments were cloned in the pGEM®-T Easy Vector System (Promega Biotech Ibérica, SL, Madrid, Spain) and transformed into competent cells of *E. coli* DH5α (Promega). Transformation was confirmed through PCR using the M13F and M13R primers as described (Alcántara-de la Cruz et al., 2016b). The colonies containing the length of the fragment were sequenced by the STABVIDA sequencing service (Caparica, Portugal). Five biological samples were used per accession providing 15 clones in all for each one. The quality and assembly of cDNA sequences and consensuses were determined employing the programs of SeqMan Pro^TM^ versión 11(DNASTAR; Wisconsin, USA) and Geneious® versión 8.1.8 (Biomatters Ltd, Auckland, New Zealand). The multiple sequences were aligned by means of the Muscle algorithm incorporated into SeqMan Pro versión 11.

### Data analysis

Dose-Response and EPSPS enzyme activity data were subjected to non-linear regression analysis (Seefeldt et al., 1995; Burgos et al., 2013) using a three-parameter log-logistic equation (Equation 1) to determine the glyphosate dose causing 50% reduction in above-ground vegetative biomass (GR_50_), 50% mortality (LD_50_), and inhibition of EPSPS activity by 50% (I_50_).

(1) $$Y=\{[(d)\;/\;(1+{\left(x/g\right)}^{b})]\}$$

Where *Y* is the EPSPS activity, survival or above-ground biomass at herbicide *x* dose, *d* is the coefficient corresponding to the upper asymptote, *b* is the slope of the curve, and *g* is the herbicide rate at the point of inflection halfway (i.e., LD_50_, GR_50_, I_50_).

Regression analyses were conducted using the *drc* package (Ritz et al., 2015) for the statistical environment R (R 3.2.4; R Core Team, 2015). Resistance indices were computed as R-to-S GR_50_ LD_50_, or I_50_ ratios. To test for a common GR_50_, LD_50_, or I_50_ for R and S accessions, i.e., Resistance Index equals to 1, a lack-of-fit test was used to compare the model consisting of curves with accessions-specific *g* values with a reduced model with common g (Ritz et al., 2015).

Analysis of variance (ANOVA) was conducted using Statistix 9.0 (Analytical Software, USA) to test for differences between R and S accessions in shikimate accumulation at 1000 μM glyphosate in the leaf segment; and proportion of the different glyphosate metabolites; proportion of applied ^14^C-glyphosate taken up by leaves, and proportions of absorbed ^14^C-glyphosate remaining in the treated leaf, translocated to roots and to the rest of the plant at 96 HAT; and basal enzyme activity. Percentage data were previously transformed (arcsine of the square root) to meet model assumptions. Model assumptions of normal distribution of errors and homogeneous variance were graphically inspected. When needed, differences between means were separated using the Tukey HSD test.

## Results

### Physiological studies

**Figure 1:**
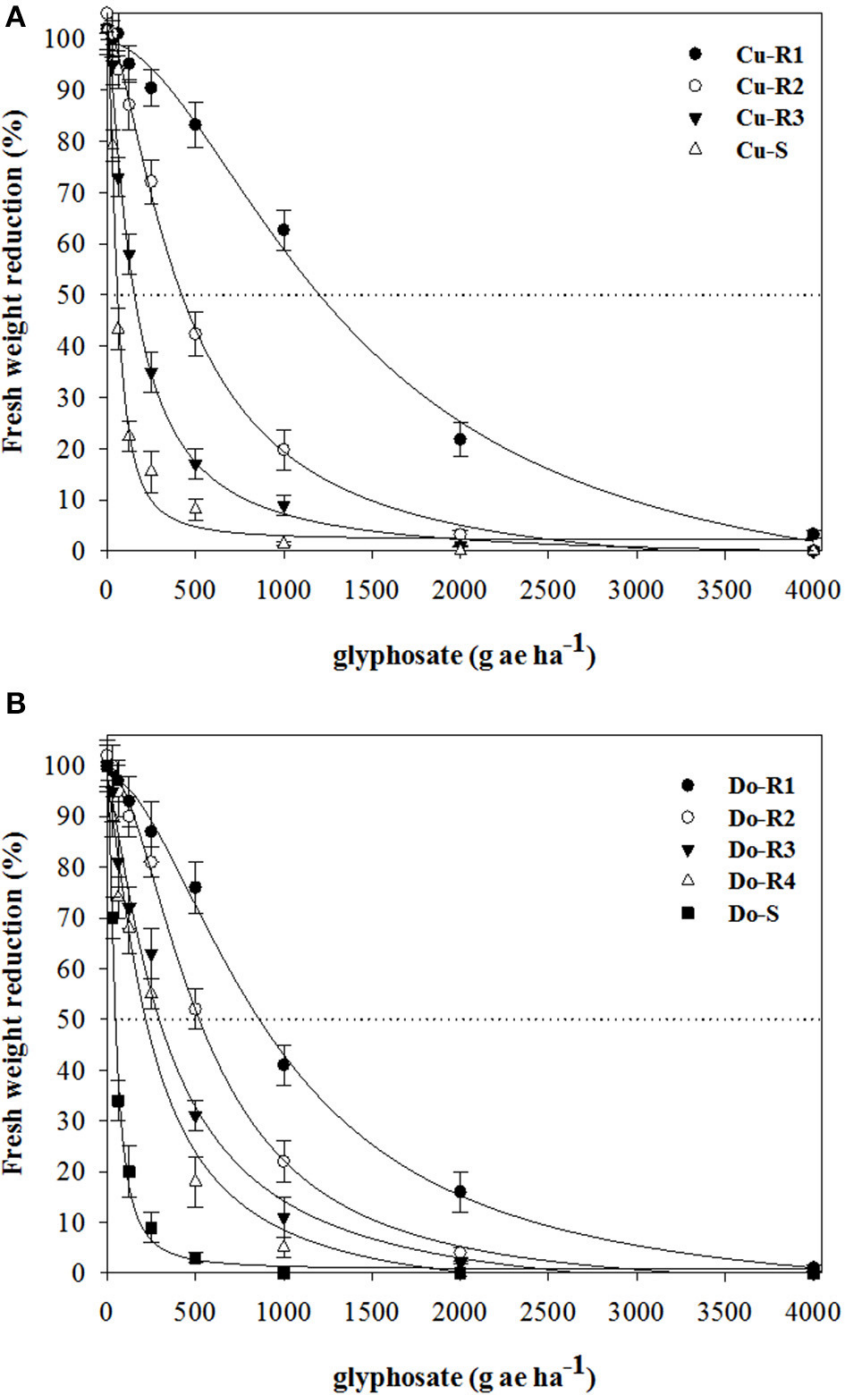
**Shoot biomass in glyphosate-resistant and susceptible *P. hystherophorus* accessions from Cuba (A)** and Dominican Republic **(B)** 30 days after treatment. Symbols denoted mean (*n* = 10) ± standard errors of the mean.

**Figure 2:**
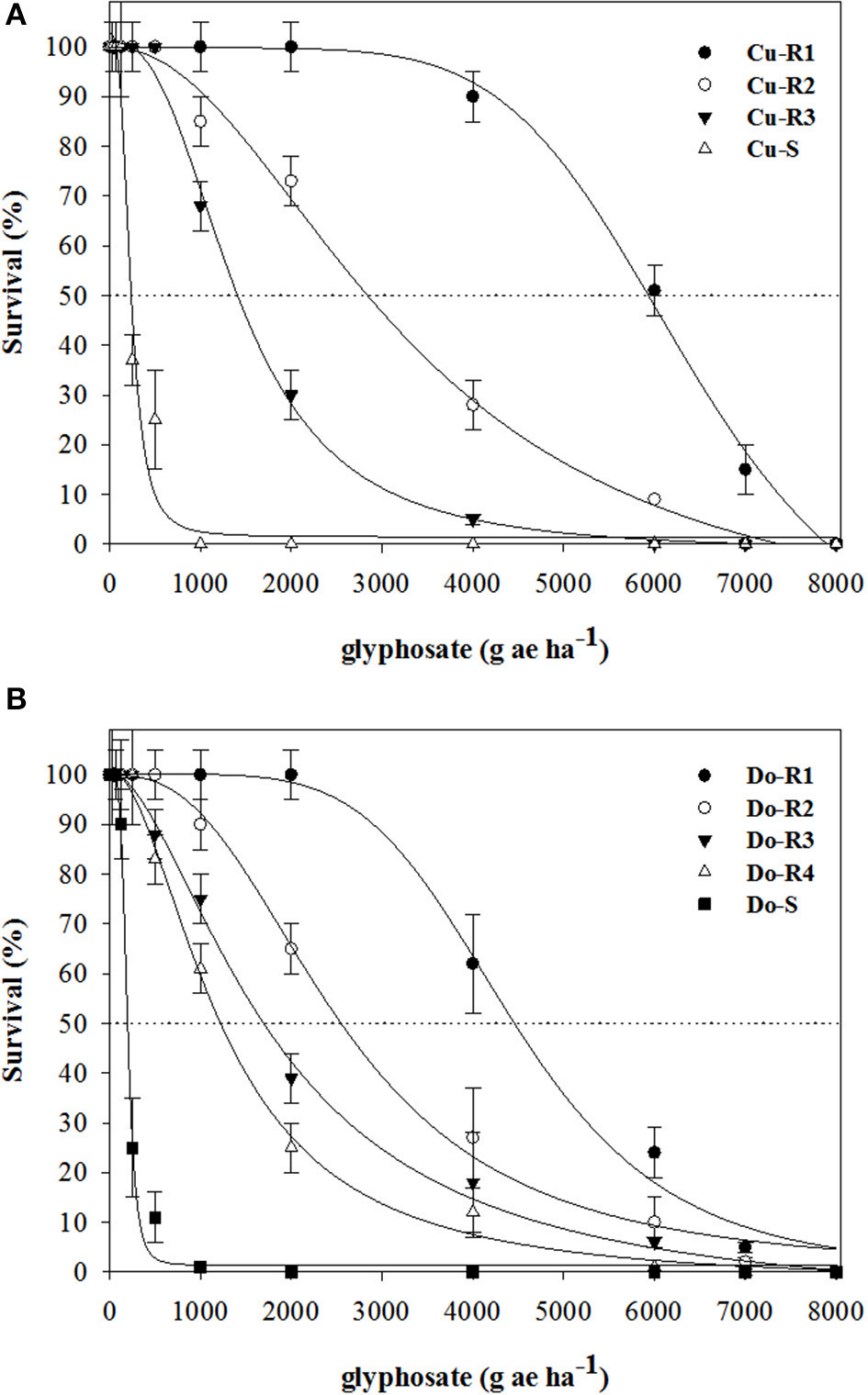
**Survival plants in glyphosate-resistant and susceptible *P. hystherophorus* accessions from Cuba (A)** and Dominican Republic **(B)** 30 days after treatment. Symbols denoted mean (*n* = 10) ± standard errors of the mean.

Dose-response assays showed the existence of the first case of glyphosate-resistant weeds in the Caribbean (Cuba and Dominican Republic). The two susceptible weeds (Cu-S and Do-S) had similar susceptibility levels (Figures 1, 2; Table 2). The *P. hyterophorus* accessions from Cuba island had resistance index (RI) values (based on the GR_50_ and LD_50_ values) that ranged from 2.7 to 24.6, and 6.1 to 27.5 fold resistance, respectively, while on Dominican Republic island values were between 5.4 to 20, and 6.3 to 22.7 fold resistance, respectively (Table 2).

**Table 2:** **Parameters of the log-logistic equations used to calculate the glyphosate rates required for 50% survival (LD_50_) and reduction fresh weight (GR_50_) of the different accessions of *P. hyterophorus* from Cuba and Dominican Republic**.

**Accessions**	**Survival^a^ (%)**						**Fresh weight reduction^b^ (%)**					
	***d***	***b***	***R*^2^**	**LD_50_ (g ae ha^−1^)**	**RI**	***p***	***d***	***b***	***R*^2^**	**GR_50_ (g ae ha^−1^)**	**RI**	***p***
Cu-R1	99.8	6.1	0.98	6364 ± 122	27.5	<0.0001	99.4	1.8	0.99	1370 ± 191	24.5	<0.0001
Cu-R2	98.9	2.9	0.99	2794 ± 90	12.0	<0.0001	103.0	1.5	0.95	437 ± 28	7.8	<0.0001
Cu-R3	100.9	2.6	0.99	1415 ± 55	6.1	<0.0001	103.3	1.3	0.96	151 ± 13	2.7	0.003
Cu-S	102.7	3.1	0.97	232 ± 23	–	–	103.2	1.7	0.98	56 ± 6	–	–
Do-R1	100.1	5.1	0.96	4456 ± 76	22.7	<0.0001	98.2	1.8	0.98	939 ± 25	20.0	<0.0001
Do-R2	99.9	2.7	0.98	2550 ± 92	13.0	<0.0001	99.6	1.8	0.99	547 ± 30	11.6	<0.0001
Do-R3	100.7	1.7	0.99	1821 ± 63	9.3	<0.0001	97.9	1.3	0.99	339 ± 27	7.2	<0.0001
Do-R4	100.9	1.9	0.99	1242 ± 65	6.3	<0.0001	96.4	1.3	0.96	255 ± 33	5.4	<0.0001
Do-S	100.5	4.5	0.97	196 ± 8	–	–	100.6	1.7	0.98	47 ± 4	–	–

a For Y = {(d) / [1 + (x/ LD_50_) exp b]} Where Y is the survival expressed as a percentage of the untreated control, d is the coefficient corresponding to the upper asymptote, b is the slope of the curve in LD_50_, LD_50_ is the herbicide rate at the point of inflection halfway, and x is the herbicide dose.

b For Y = (d) / [1 + (x/ GR_50_) exp b] Where Y is the above-ground weight expressed as a percentage of the untreated control, d is the coefficient corresponding to the upper asymptote, b is the slope of the curve in GR_50_, GR_50_ is the herbicide rate at the point of inflection halfway, and x is the herbicide dose.

**Figure 3:**
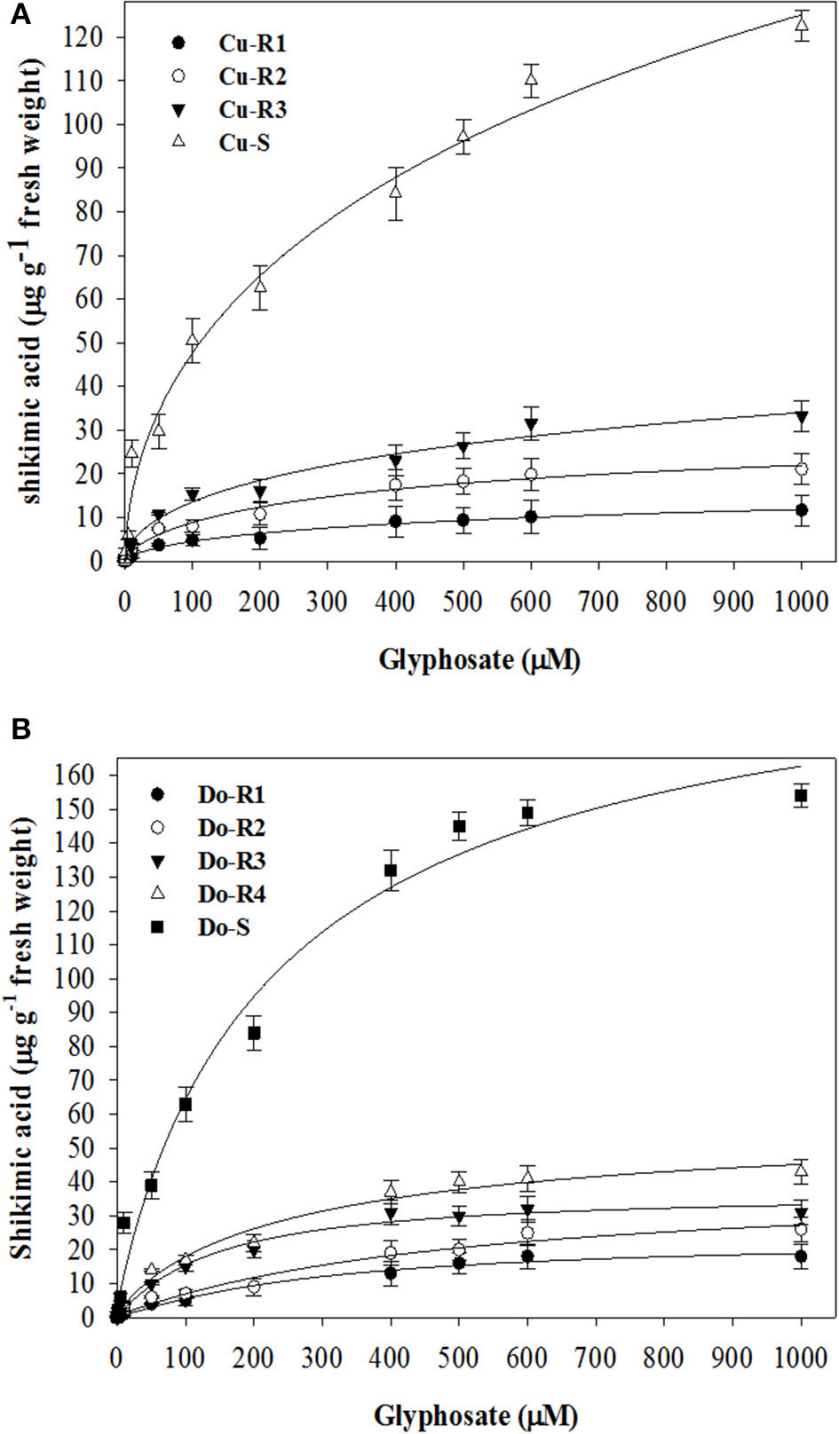
**Shikimic acid accumulation in leaf segments of plants from Cuba (A)** and Dominican Republic **(B)** accessions of *P. hysterophorus*. Symbols denoted mean (*n* = 3) ± standard errors of the mean.

The fact that plants treated with glyphosate increase shikimic acid accumulation in leaf disks due to the inhibition of EPSPS activity led us to carry out the experiment depicted in Figures 3A,B. Considering the values obtained *in vivo* (GR_50_ and LD_50_) and the shikimic acid accumulation in leaf disks at 1000 μM of glyphosate, the resistance order of the *P. hystherophorus* accessions was Cu-R1 ≥ Do-R1 > Do-R2 > Cu-R2 > Do-R3 > Do-R4 > Cu-R3 >> Cu-S ≥ Do-S. There were significant differences at 1000 μM glyphosate between R and S accessions of Cuba (*p* = 0.0013, *DF* = 3, *n* = 12) and Dominican Republic (*p* = 0.0008, *DF* = 4, *n* = 15).

There were marked differences in glyphosate absorption between the resistant and susceptible glyphosate *P. hysterophorus* accessions at 96 h after treatment (HAT) (*p* = 0.0001, *DF* = 8, *n* = 45) (Table 3). All accessions obtain maximum absorption at 96 HAT, and the two susceptible accessions absorbed an average of 80.5%, while the resistance accessions absorbed an average of 59.2% of ^14^C-glyphosate which was recovered.

**Table 3:** **^14^C-glyphosate absorption (% of recovered radioactivity) and translocation (% of absorbed radioactivity) in the different *P. hysterophorus* accessions at 96 h after treatment (HAT)**.

**Accessions**	**Absorption^a^ (**p** = 0.0001, **DF** = 8, **n** = 45)**	**Translocation**		
		**Treated leaf (**p** = 0.0003, **DF** = 8, **n** = 45)**	**Rest of shoot (**p** = 0.0001, **DF** = 8, **n** = 45)**	**Root (**p** = 0.0004, **DF** = 8, **n** = 45)**
Cu-R1	59.3 ± 4.9 BC	77.9 ± 5.7 AB	12.1 ± 2.1 BCD	10.0 ± 2.3 BC
Cu-R2	60.2 ± 2.1 BC	82.4 ± 4.2 A	9.3 ± 1.9 D	8.3 ± 3.4 BCD
Cu-R3	56.8 ± 3.9 C	80.1 ± 3.9 AB	15.7 ± 3.4 B	4.2 ± 1.2 D
Cu-S	82.2 ± 6.7 A	35.5 ± 2.3 C	41.6 ± 6.2 A	22.9 ± 4.8 A
Do-R1	63.1 ± 6.8 B	78.3 ± 6.7 AB	10.5 ± 2.7 CD	11.2 ± 2.1 B
Do-R2	55.9 ± 7.8 C	79.3 ± 3.4 AB	16.2 ± 4.9 B	4.5 ± 1.4 D
Do-R3	60.4 ± 3.7 BC	75.6 ± 5.1 B	14.1 ± 3.8 BC	10.3 ± 3.8 B
Do-R4	58.4 ± 2.3 BC	81.4 ± 6.3 A	12.7 ± 4.3 BCD	5.9 ± 2.7 CD
Do-S	78.8 ± 5.6 A	39.1 ± 1.9 C	37.8 ± 2.3 A	23.1 ± 5.6 A

a Over 95% of the total radioactivity applied was recovered.

Mean value (n = 5) ± standard error. Means on a same column followed by the same letter were not significantly different at α = 0.05.

Translocation assays suggest marked differences at 96 HAT between the Cu-S and Do-S accessions compared to the Cu-R1, Cu-R2, Cu-R3, Do-R1, Do-R2, Do-R3, and Do-R4 ones in treated leaf (*p* = 0.0003, *DF* = 8, *n* = 45), rest of the shoots (*p* = 0.0001, *DF* = 8, *n* = 45), and root (*p* = 0.0004, *DF* = 8, *n* = 45) (Table 3). There were no significant differences in translocation between the two susceptible accessions (Cu-S and Do-S) from Caribbean Islands. But there were small significant differences in the resistant accessions (Cu-R1, Cu-R2, Cu-R3, Do-R1, Do-R2, Do-R3, and Do-R4). Nonetheless, the high amount of ^14^C-glyphosate in each resistant accession remained in the treated leaf. Due to differences in levels of glyphosate resistance between the *P. hysterophous* resistant accessions, we suspect that other mechanisms could be involved (Tables 2, 3, Figure 3).

### Biochemical studies

Previous tests demonstrated that the highest glyphosate translocation and metabolism was reached at 96 HAT in the *P. hysterophorus* accessions (unpublished data). There were significant differences at 96 HAT in glyphosate metabolism levels between accessions (*p* = 0.0014, *DF* = 8, *n* = 36). Glyphosate levels decreased, whereas glyphosate metabolites (AMPA, glyoxylate and sarcosine) increased at 96 HAT in the Cu-R1, Do-R1, Do-R2, Cu-R2, and Do-R3 accessions. Higher glyphosate levels remained in the Cu-R3 and Do-R4 (low resistance), and very high one in the Cu-S and Do-S (susceptible) accessions. In these last accessions, sarcosine was not detected (Table 4). These results can also explain the low level of resistance of the accession (Cu-R3 and Do-R4) with a single resistance mechanism, while the other glyphosate resistant accessions have at least two mechanisms (Tables 3, 4).

**Table 4:** **Glyphosate metabolism expressed as a percentage of total glyphosate and its metabolites in *P. hystherophorus* susceptible and resistant-glyphosate accessions at 96 HAT**.

**Accessions**	**Glyphosate (**p** = 0.0014, **DF** = 8, **n** = 36)**	**Metabolites**		
		**AMPA (**p** = 0.0003, **DF** = 8, **n** = 36)**	**Glyoxylate (**p** = 0.0001, **DF** = 8, **n** = 36)**	**Sarcosine (**p** = 0.0002, **DF** = 8, **n** = 36)**
Cu-R1	9.80, 1.70D	60.54, 1.32B	18.14, 0.32C	11.52, 0.96A
Cu-R2	21.12, 0.93C	55.31, 1.57B	20.80, 0.51AB	2.77, 0.31E
Cu-R3	73.42, 3.63B	26.14, 0.26C	0.44, 0.02E	ND
Cu-S	91.82, 4.81A	7.68, 0.33E	0.50, 0.02E	ND
Do-R1	11.83, 0.74D	58.94, 2.79B	21.74, 0.97A	7.49, 0.27C
Do-R2	11.37, 0.80D	64.70, 2.93A	18.54, 0.83C	5.39, 0.15D
Do-R3	9.56, 0.72D	60.95, 2.71B	20.36, 0.94B	9.13, 0.53B
Do-R4	71.21, 1.06B	20.05, 2.20D	7.28, 0.93D	1.01, 0.71F
Do-S	90.68, 4.39A	8.86, 1.06E	0.46, 0.03E	ND

Mean value (n = 4) ± standard error. Means on a same column followed by the same letter were not significantly different at α = 0.05.

ND, non-detected; AMPA, aminomethylphosphonic acid.

**Figure 4:**
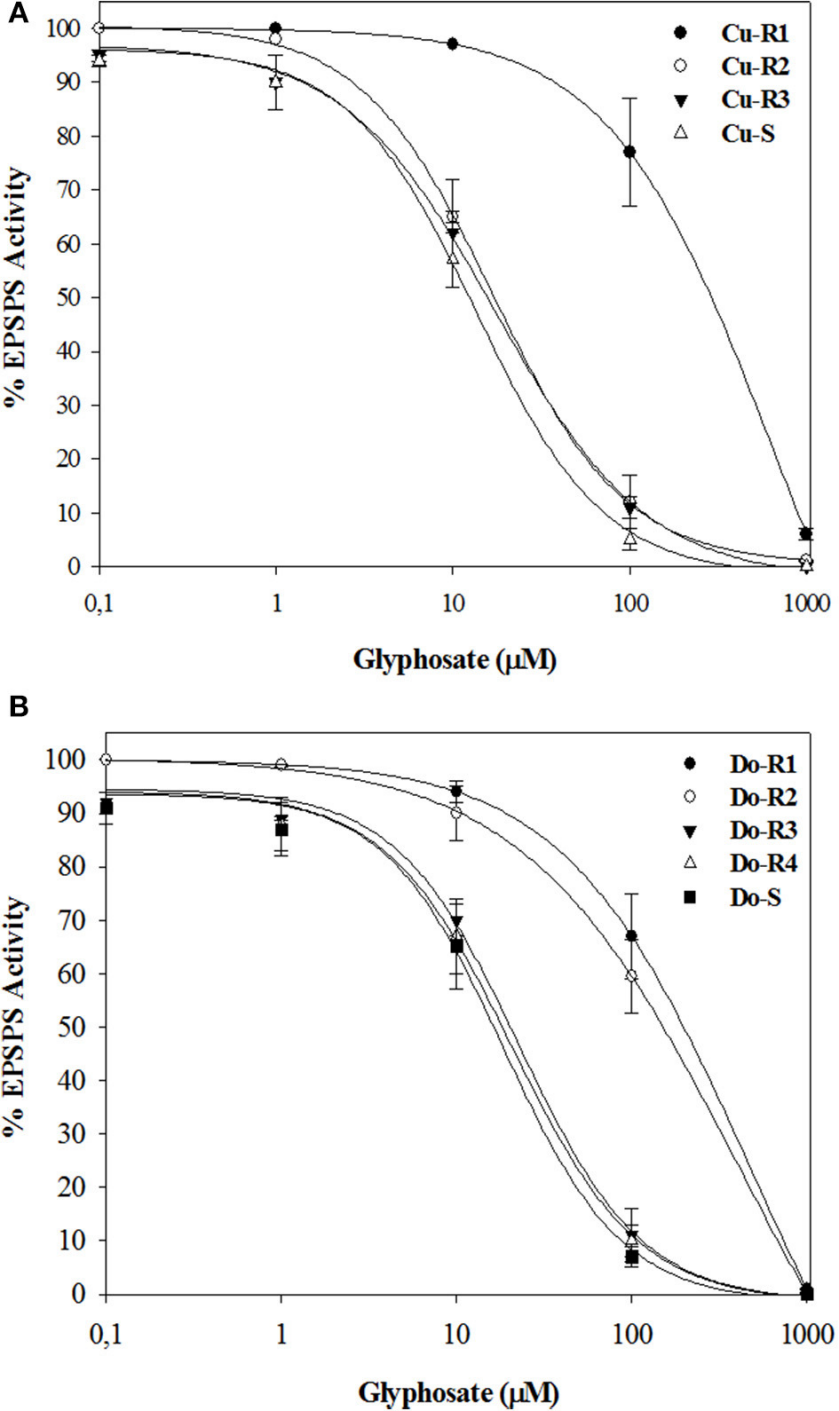
**EPSPS enzyme activity expressed as percentage of the untreated control in leaf extracts of plants from Cuba (A)** and Dominican Republic **(B)** accessions of *P. hysterophorus*. Symbols denoted mean (*n* = 3) ± standard errors of the mean.

The EPSPS enzymes of all the accession plants were inhibited by glyphosate. The I_50_ (herbicide dose which reduces the enzyme activity to 50%) values were different in all accessions, ranging between approximately 47.65 in Cu-R1, 25.2 in Do-R1, 22.1 in Do-R2, 1.4 in Cu-R2, 1.2 in Do-R3, 1.2 in the Cu-R3, and 1.1-fold resistance in Do-R4 accessions relative to their susceptible accession, respectively (Figure 4, Table 5). These results were in accordance with the *in vivo* resistance level shown for the different accessions, and suggest that multiple mechanisms in the target-site could be expressed in these accessions.

**Table 5:** **Parameter estimates of the equation used to calculate the sensitivity of EPSPS enzyme activity to glyphosate in extracts from leaf tissue of the different accessions of *P. hyterophorus* from Cuba and Dominican Republic**.

**Accessions**	***d***	***b***	***R*^2^**	**I_50_ (μM)^a^**	**RI**	***P***
Cu-R1	100.1	0.9	0.97	646.2 ± 35.8	47.6	<0.0001
Cu-R2	99.8	0.8	0.96	18.9 ± 1.4	1.4	0.1902
Cu-R3	97.0	1.0	0.99	17.4 ± 2.8	1.2	0.2186
Cu-S	96.2	1.2	0.98	13.6 ± 2.2	–	–
Do-R1	100.0	0.8	0.99	468.1 ± 22.0	25.2	<0.0001
Do-R2	100.4	0.7	0.99	410.7 ± 26.1	22.1	<0.0001
Do-R3	94.5	1.2	0.98	22.6 ± 1.5	1.2	0.3714
Do-R4	94.0	1.2	0.96	20.8 ± 6.1	1.1	0.6042
Do-S	93.6	1.2	0.99	18.5 ± 5.7	–	–

a For Y = {(d) / [1 + (x/ I_50_) exp b]} Where Y is the EPSPS activity, d is the coefficient corresponding to the upper asymptote, b is the slope of the curve in I_50_, I_50_ is the herbicide rate at the point of inflection halfway, and x is the herbicide dose.

**Figure 5:**
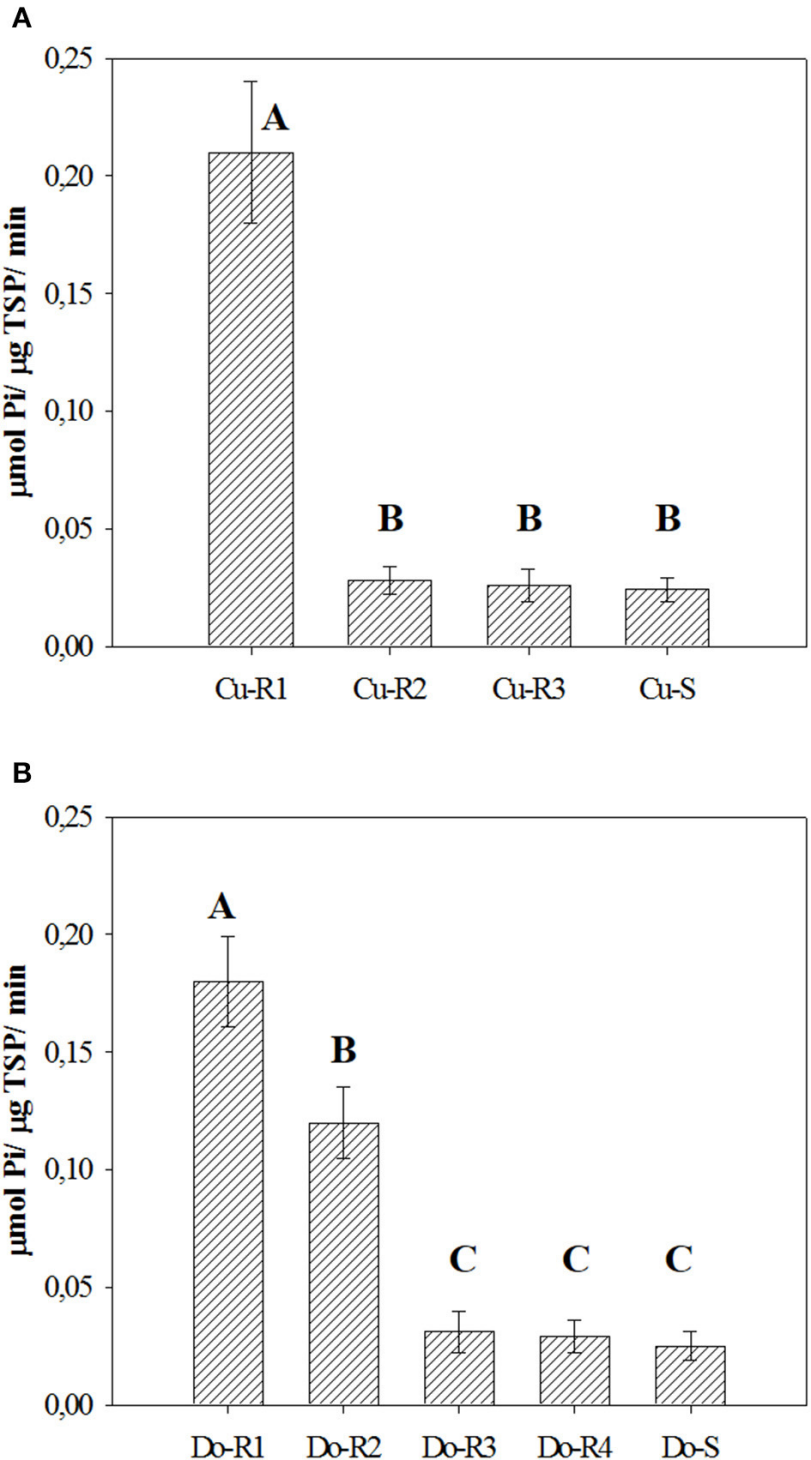
**Basal EPSPS activity for glyphosate-susceptible and resistant from Cuba (A)** and Dominican Republic **(B)** accessions of *P. hysterophorus*. Vertical bars are ± standard errors of the mean. Means by the same letter were not significantly different at α = 0.05.

The basal activity of EPSPS enzyme (without glyphosate) in the resistant accessions was between 0.026 and 0.21 μmol μg^−1^ protein min^−1^, while the susceptible accessions (Cu-S and Do-S) were lower with 0.024 and 0.025 μmol μg^−1^ protein min^−1^, respectively (Figure 5). There were market differences between accessions in both Cuba (*p* = 0.0001, *DF* = 3, *n* = 12), and Dominican Republic (*p* = 0.0002, *DF* = 4, *n* = 15). The Cu-R1, Do-R1, and Do-R2 exhibited 8.8, 7.2, and 4.8-times higher basal enzyme activities than their susceptible accessions, respectively. For Cu-R2, Do-R3, Do-R4, and Cu-R3 accessions the values were similar to those found for their susceptible accessions, respectively.

### Molecular studies

**Figure 6:**
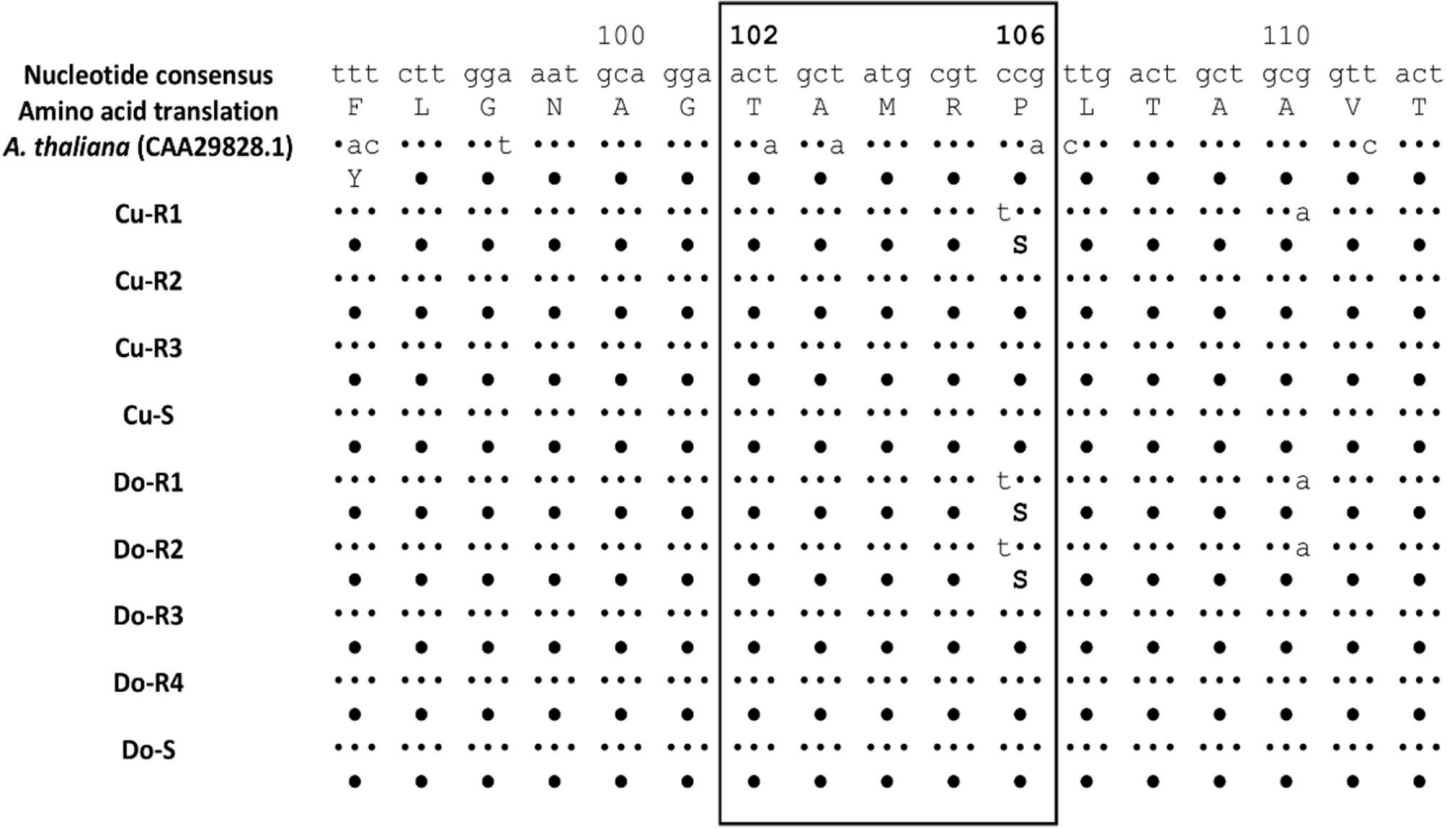
**Partial protein sequence alignment of the EPSPS gene of resistant and susceptible *P. hysterophorus* plants**. The box comprising the region of Thr-102 and Pro-106 point mutations associated to confer glyphosate resistance. The points indicate homology between the different sequences.

A total of 462 bp of the EPSPS gene of *P. hysterophorus* plants of resistant and susceptible accessions were sequenced. The fragments were aligned and numbered based on a published EPSPS sequence of *Arabidopsis thaliana* (L.) Heynh. (GenBank: CAA29828.1). The resistant accessions of *P. hysterophorus* Cu-R1 from Cuba, and Do-R1 and Do-R2 from Dominican Republic, showed an amino acid substitution at position 106 consisting of a Proline to Serine (Figure 6).

## Discussion

*P. hysterophorus* is universally recognized for its widespread distribution and high seed production, commonly known as the parthenium weed. Parker (1989) identified two biotypes with different flowering patterns in Mexico (Caribbean area), and they were genetically distinct biotypes (Clermont and Toogoolawah). Moreover, Hanif et al. (2011) found that these two biotypes differed in their morphology and reproductive behavior; in particular, the Toogoolawah biotype shows a greater tendency toward self-pollination, but these biotypes can also present out-crossing. It makes sense that it would reproduce prolifically and that higher resistance levels due to accumulation of multiple mechanisms, by multiple crossings, would proliferate within populations (Table 6).

**Table 6:** **Summary of glyphosate resistance mechanisms accumulated by *P. hysterophorus* accessions studied in this work**.

**Accessions**	**GR_50_^a^**	**LD_50_^a^**	**Absorption and translocation**	**Glyphosate metabolism**	**Enhanced EPSPS basal activity^b^**	**EPSPS (I_50_^b^)**	**Pro106Ser**
Cu-R1	1370	6364	Low	High	Yes	High	Yes
Cu-R2	437	2794	Low	High	No	Low	No
Cu-R3	151	1415	Low	Medium	No	Low	No
Cu-S	56	232	High	Low	No	Low	No
Do-R1	939	4456	Low	High	Yes	High	Yes
Do-R2	547	2550	Low	High	Yes	High	Yes
Do-R3	339	1821	Low	High	No	Low	No
Do-R4	255	1242	Low	Medium	No	Low	No
Do-S	47	196	High	Low	No	Low	No

a glyphosate g ae ha^−1^;

b glyphosate μM.

Glyphosate has been used repeatedly in perennial crop areas and fallow fields in the Caribbean Islands for many years to manage *P. hysterophorus* and other troublesome weeds. However, using glyphosate alone without any additional alternative and/or IWM (Integrated Weed Management) led to the emergence of glyphosate-resistant weeds early in the second decade of the 21st century (Tables 1, 2). Herbicide response between different locations depends on local ecological factors, such as a variation in soil type, tillage practices, types of crops, fertilizers, etc., (Shaner and Beckie, 2014; Jussaume and Ervin, 2016). Our results showed different glyphosate resistance levels between the *P. hysterophorus* accessions. This differences could be addressed to the use of different glyphosate formulations and dose rate, the application technique (manual or mechanical) employed by farmers, and the agro environment conditions (Neve et al., 2014; Renton et al., 2014; Jussaume and Ervin, 2016; Matzrafi et al., 2016; Owen, 2016). It has been shown that an increase in the relative humidity and temperature increases the glyphosate absorption, translocation, and toxicity in many weed species (Ge et al., 2011; Hatterman-Valenti et al., 2011; Vila-Aiub et al., 2012; Santos et al., 2016). This research also revealed that the low GR_50_ and LD_50_ values for the susceptible accessions showed that glyphosate has been a very effective tool for farmer for over 15 years, as has been shown in *P. hysterophorus* from Colombia, Dominican Republic, and Florida (Fernandez, 2013; Rosario et al., 2013; Jimenez et al., 2014).

Plants with low levels of GR_50_ and LD_50_ are related to an increased inhibition of EPSPS activity and a greater accumulation of shikimic acid (Shaner et al., 2005; Gaines et al., 2010; Fernández et al., 2015). High levels of resistance (RI) and low shikimic acid accumulation observed in the different *P. hystherophorus* accessions were consistent with those of plants which have acquired resistance to the addition of more than one NTSR and/or TSR mechanisms, as has been shown in dicotyledonous weed species such as *Amaranthus tuberculatus* (Nandula et al., 2013), *Conyza sumatrensis* (González-Torralva et al., 2014), and several grass weed species (Michitte et al., 2007; de Carvalho et al., 2012; Fernández et al., 2015).

According to Shepherd and Griffiths (2006), a cuticular wax layer provides a protective barrier for a wide range of abiotic stresses (pesticide). Resistant and tolerant plants have displayed a cuticle containing a massive amount of epicuticular wax which forms a nonuniform 3D cover as has been revealed by scanning electron micrographs (De Prado et al., 2005; Wang and Liu, 2007; Rojano-Delgado et al., 2012; Alcántara-de la Cruz et al., 2016a). The limited glyphosate absorption by the resistant *P. hysterophorus* accessions was likely to have been due to differences in outer leaf surfaces. Different translocation can be explained by ^14^C-glyphosate and/or its metabolite accumulation in the tips of the resistant treated leaves, while ^14^C was removed from the susceptible treated leaves (Table 3). Since the first case of glyphosate resistance was detected in a population of *Lolium rigidum* in Australia (Powles et al., 1998), both previously mentioned mechanisms were considered responsible for this resistance (Wakelin et al., 2004; Michitte et al., 2007; Preston and Wakelin, 2008; de Carvalho et al., 2012; González-Torralva et al., 2012, 2014; Nandula et al., 2013; Fernández et al., 2015). Subsequent studies in the main dicot and monocotyledonous glyphosate-resistant weeds seem to have demonstrated that the main NTSR mechanism involved in their resistance is due to a lesser glyphosate absorption and/or -translocation (Feng et al., 2004; Michitte et al., 2007; de Carvalho et al., 2012; González-Torralva et al., 2012, 2014; Vila-Aiub et al., 2012; Nandula et al., 2013; Adu-Yeboah et al., 2014).

In some plants, the glyphosate degradation to glyoxylate and AMPA is carried out by a glyphosate oxidoreductase (GOX), and the glyphosate degradation to sarcosine and inorganic phosphate by a C–P lyase. These steps have been reported by some authors such as Liu et al. (1991); Komoba et al. (1992); Saroha et al. (1998); Al-Rajab and Schiavon (2010), and Duke (2012) among others. However, only a few works unify these two degradation pathways to explain the glyphosate metabolism in leguminous plants and weeds (de Carvalho et al., 2012; Rojano-Delgado et al., 2012). Some authors consider that metabolism has a low contribution to the resistance or, even more, that it is nonexistent (Saroha et al., 1998; Feng et al., 2004; Duke, 2012; Sammons and Gaines, 2014). However, the fact is that this mechanism involves a decrease in the concentration of the herbicide glyphosate around the target-site, diminishing the EPSPS inhibition rate (Duke, 2012; Sammons and Gaines, 2014; Alcántara-de la Cruz et al., 2016a). The GOX gene that encodes the glyphosate metabolizing enzyme glyphosate oxidoreductase was cloned from *Achromobacter* sp. *strain* LBAA (Barry et al., 1994). Neither plant GOX nor the gene(s) encoding it have been isolated or elucidated. A plant gene encoding GOX might be useful in genetically engineering crops and weed resistance development (Duke, 2012; Rojano-Delgado et al., 2012). Some researchers have proposed additive effects of concurrent glyphosate resistance mechanisms in the same weed species (Gaines et al., 2010; Yu et al., 2010; Bostamam et al., 2012; Rojano-Delgado et al., 2012), which would explain the difference in the resistance between accessions keeping the same percentage of metabolic degradation (Table 6). However, genetic basic controlling absorption/translocation and/or metabolism including genes involved have not been identified so far (Yuan et al., 2006; Delye, 2013; Délye et al., 2013). This could be a highly promising research area in the future.

Taking into account these results, resistance could be associated with target enzyme overexpression. Some species as ryegrass (Yu et al., 2007; Dayan et al., 2012) have shown differences in the basal EPSPS enzyme activity as a consequence of the EPSPS gene overexpression. However, in the *L. perenne* spp. *multiflorum* population from Arkansas, no differences were observed in the I_50_ values, which could be explained as a lack of effective mutations in the binding site of the enzyme (Salas et al., 2015). In our case, some accessions are candidates to possessing an effective mutation (Figure 6, Table 6) or a possible EPSPS overexpression, explaining their high resistance to glyphosate compared to other accessions. We are aware of that fact, and effective research is currently in progress to characterize the EPSPS overexpression resistance mechanism involving these accessions.

Results reported here are in agreement with previous works, in which the Proline to Serine substitution was found to confer glyphosate resistance in other weed species such as *A. tuberculatus, C. sumatrensis, Echinochloa colona*; *L. perenne* spp. *multiflorum* and *L. rigidum* (Bostamam et al., 2012; González-Torralva et al., 2012, 2014; Nandula et al., 2013; Fernández et al., 2015; Han et al., 2016). However, mutations in the Pro-106 position generally provide only a low level (2–4-fold) of glyphosate resistance (Kaundun et al., 2011). Here, *P. hysterophorus* accessions that presented Pro-106 mutation had a resistance factor of >12. These three accessions (Cu-R1, Do-R1, and Do-R2) were more highly resistant to glyphosate as a result of showing different concurrent resistance mechanisms, including reduced absorption and translocation, glyphosate metabolism, and EPSPS gene mutation.

In some species, at least more than one glyphosate resistance mechanism have been reported, such as *A. tuberculatus* (Nandula et al., 2013), *L. rigidum* (Bostamam et al., 2012), *L. perenne* spp. *multiflorum* (González-Torralva et al., 2012), and *L. perenne* (Ghanizadeh et al., 2015) populations which exhibited a mutation in Pro-106 position, and a reduced translocation. Besides, other species such as *Digitaria insularis* presented a pool of mechanisms (absorption, translocation, metabolism, and EPSPS gene mutation; de Carvalho et al., 2012). The involvement of several resistance mechanisms is evident when looking at the resistance levels of accessions Cu-R2, Cu-R3, Cu-R4, Do-R3, Do-R4, and Do-R5 of *P. hysterophorus*, which did not show any mutation in the Pro-106 position. This is the first time that a mutation in the target-site has been reported in glyphosate-resistant *P. hysterophorus*.

In summary, we have confirmed resistance to glyphosate in different *P. hysterophorus* accessions harvested in the Caribbean Islands. Their resistance levels depend on the different resistance mechanisms (NTSR and TSR) that are accumulated by these accessions (Table 6), due to increasing selection pressure and out-crossing. The evolution of multiple mechanisms found in this resistance species is worrying. The farmers should implement manage practices such as the use of cover crops, which prevent soil erosion and allow the use of grazing, as well as the use of other non-selective herbicides in an integrated weed management (IWM) to facilitate the reduction and suppression of herbicide-resistant accessions.

## Author contributions

EB, PF, and RD performed the glyphosate plant dose-response and shikimic acid accumulation. EB, PF, FB, and RD carried out the EPSPS activity assays. EB, PF, and RD did the ^14^C-glyphosate absorption/translocation, and metabolism study. FB performed the EPSP synthase gene sequencing.

## Funding

This work was funded by AGL2013-48946-C3-1-R and AGL2016-78944-R projects (Spain).

### Conflict of interest statement

The authors declare that the research was conducted in the absence of any commercial or financial relationships that could be construed as a potential conflict of interest.
